# Supplementary material for: Trigeminal nerve stimulation (TNS) for children with attention deficit/hyperactivity disorder and fetal alcohol spectrum disorder: Feasibility study protocol
Source: PLoS One. 2025 Aug 29;20(8):e0330986. doi: 10.1371/journal.pone.0330986 (PMC12396707; doi:10.1371/journal.pone.0330986)
Supplement: S2 File — (PDF) [file pone.0330986.s002.pdf]

Protocol Title: Trigeminal Nerve Stimulation for Children with Prenatal Alcohol Exposure

Protocol Version: 1.0

Protocol Date: 2/5/2025

Principal Investigator: Joseph O'Neill, PhD

Project Summary:

Recently, it was demonstrated at UCLA that trigeminal nerve stimulation (TNS) is safe and effective in treating pediatric ADHD. TNS is a noninvasive, non-pharmaceutical, minimal-risk, low-current neurostimulatory therapy for ADHD. TNS is now FDA-cleared for children with ADHD not currently taking ADHD medication. The FDA-clearance makes no distinction with respect to the etiology of the ADHD, but the possible influence of etiology on TNS response has not yet been systematically investigated. One of the major etiologies of ADHD is prenatal alcohol exposure (PAE). To our knowledge, TNS has never been tested systematically tested in children whose ADHD is likely due to PAE. Here we propose the first clinical trial of TNS for children with ADHD associated with PAE. There is a pressing need for such a study as ADHD is very common in PAE, ADHD symptoms are among the most disabling in PAE, and children with PAE frequently respond poorly to conventional pharmaceutical treatments (i.e, psychostimulants) for ADHD. PAE, moreover, is a severe, life-long neurodevelopmental disorder. It afflicts hundreds of thousands of children in the US and worldwide and treatment options are few. Therefore, it is incumbent to explore every reasonable prospective treatment in children with PAE, particularly TNS. Based on our prior neuroimaging studies of ADHD in PAE, we are optimistic that TNS will be efficacious in relieving ADHD symptoms, as we have seen abnormalities in the brains of children with ADHD due to PAE in the same brain regions that TNS is thought to act upon in EEG studies. But there is a chance TNS might not be effective, therefore it must be tested explicitly. Children with PAE are very frequently prescribed psychotropic medication, to the point where it would not be feasible to recruit a sufficiently large sample size within the study term if medicated children were excluded. Therefore, this study will include children who are on ADHD medication. Since some subjects will be medicated, the study overall will **not** be within current labelling of the TNS device. Therefore, as per standard UCLA IRB policy, based on his Initial Assessment, the PI made a determination that the study TNS device is a non-significant risk (NSR) device. The IRB has agreed with this determination. Rather than "use of an FDA-cleared device for an approved indication" it has classified the research as "investigational use of an NSR device". This classification was granted for the Generation 1.0 TNS device which we plan to use in this study. The manufacturer, however, will phase-out the Gen 1 device and replace it with a Generation 2.0 TNS device as of January 2025. The Gen 2 device is highly similar to Gen 1; in particular, it delivers the same electrical stimulation waveform to the patient. The PI has also requested an NSR classification for the Generation 2.0 TNS device to use in this trial, in case it is needed to replace damaged or lost Gen 1 devices.

This IRB proposal is associated with our R61/R33 grant proposal (currently pending funding approval at NIAAA). The R61 (pilot) phase will determine whether TNS is feasible for PAE: Do children with PAE comply with TNS procedures? Does TNS have any serious side effects in these children? Is there an indication that TNS relieves symptoms of PAE? Is such relief retained in follow-up? If the pilot shows feasibility, as we expect it will, we will be in a position to apply for continued funding of an R33 phase. In that R33, we would conduct a formal double-blind sham-controlled randomized clinical trial to determine rigorously whether TNS is truly efficacious for PAE. The present IRB proposal concerns only the R61 phase. If the R33 is later funded, we will submit an amended proposal to the IRB at that time. If TNS is efficacious for PAE, the impact could be enormous. TNS could bring much needed relief to children with PAE and their families everywhere. Our proposal, furthermore, will address the need to understand the brain mechanisms by which TNS works. These are underexplored and largely unknown for ADHD or any condition treated by TNS. The few studies done to date point to three major candidate brain regions where TNS may exercise its therapeutic effects. All three are regions where

we, in prior work, detected differences between children with PAE and typically developing children in tissue properties ("endpoints") measured using multiple different varieties ("modalities") of brain MRI. These imaging findings raise our confidence that TNS will be effective for PAE. I.e., if the brain regions that produce PAE symptoms are the same as those acted upon by TNS, maybe TNS will quell those symptoms. In both phases of this clinical trial we will measure the same endpoints with the same modalities of brain MRI in children with PAE before and after TNS treatment. We will determine whether these endpoints are changed by TNS (target engagement) and/or predict TNS clinical response (prognostic marker) in individual patients in the three candidate regions or elsewhere in the brain. If successful, this proposal will help meet a long-standing urgent need for novel treatments for PAE and will help clarify the currently mysterious brain mechanisms of an emerging pediatric neurostimulatory therapy.

Background

Prenatal Alcohol Exposure (PAE) still affects more than 5% of US children despite decades of public efforts to curb ethanol consumption during pregnancy. High disability arises from the ADHD-like behavioral symptoms (hyperactivity, impulsivity, executive function deficits) that occur in 50-95% of children with PAE. In ADHD, these symptoms often improve spontaneously by adulthood. But in PAE they typically evolve into lifelong problems in attention and executive function, behavioral and emotional regulation, and adaptive function. These problems contribute to "secondary disabilities", i.e., adverse outcomes including school failure, legal trouble, substance abuse, mental illness, and suicide frequently encountered by people with PAE in adolescence and adulthood. Secondary disabilities add to the >$4 billion/year public health cost of PAE. Formal treatment studies for PAE are lacking. Psychostimulants are commonly prescribed for behavioral symptoms in both ADHD and PAE. While these drugs, however, lead to routine improvement in ADHD, their efficacy in PAE is not well established. Rather, there is much evidence that stimulants are less effective for PAE. Side effects to stimulants, moreover, may occur at higher rates in PAE. While promising evidence-based behavioral interventions for PAE have emerged at UCLA and elsewhere, children with PAE often fail to retain gains achieved with behavioral therapies. Thus, there are currently no truly effective treatment options for PAE. This proposal aims to address this urgent unmet need by conducting a pilot (R61) clinical trial of a novel treatment for behavioral symptoms of PAE.

Trigeminal nerve stimulation (TNS) was recently found safe and efficacious for core (inattention, hyperactivity) and executive-function symptoms of ADHD in unmedicated children in an open pilot and a sham-controlled double-blind randomized controlled trial (RCT) at UCLA (McGough et al. 2015,2019). The response rate (50%) was appreciable for psychiatry, comparable to the 40-60% efficacy of SRIs for major depressive disorder. While these trials led to FDA-clearance of TNS for unmedicated pediatric ADHD, patients in those trials were not screened for PAE. Given the symptom overlap of PAE and ADHD and the fact that certain putative sites of action of TNS in the brain match sites where we found imaging abnormalities in PAE, we are optimistic that TNS will also be safe and effective in alleviating the behavioral symptoms of PAE. But formal testing of this expectation is required if we are to have a firm basis for recommending TNS as a therapy for PAE. There is a chance that TNS might not be safe or effective for PAE. Stimulant drugs, as mentioned, usually work for ADHD but often not for PAE. Also, our preliminary data acquired with magnetic resonance (MR) neuroimaging has documented differences between children with ADHD-like symptoms with and without PAE in many brain regions. Such differences may influence clinical response to TNS, in positive or negative ways. A clinical trial with imaging may answer this question and help elucidate the therapeutic mechanism of TNS which (although we have some clues) is unknown for any of the conditions that it treats. Our clinical trial will test TNS for the first time in children with known PAE and will include imaging to identify brain regions that are changed by TNS and/or that predict TNS response. This trial of TNS for PAE was thus submitted under the NIAAA R61/R33 mechanism, which is designed for novel therapies, with perhaps partly understood mechanisms, holding promise for success, but with a need for rigorous testing.

The FDA-clearance for the TNS device covers only children with ADHD who are not currently taking ADHD medication. Children with ADHD associated with PAE are not specifically excluded by the labelling, nor are they specifically included. This proposal will test the TNS device in children with ADHD associated with PAE, some of whom will be taking concurrent

ADHD medication. Based on our Initial Assessment, we do anticipate additional risks due to medication and we will not ask parents to take their children off medication. Thus, TNS treatment, at least for children on medication, will be off-label. Therefore, rather than as "treatment with an FDA-cleared device within an approved indication", present research is classified as "investigational use of an NSR device".

Potential impact is very high. If TNS is safe and efficacious for PAE, this proposal will yield a much needed new therapy for managing PAE. Study data could possibly be used to apply for an expanded FDA-clearance that specifically lists PAE as an indication and that allows treatment of medicated children. Of the 49.5 million school-age children in the US, if one takes the low estimates that 1.8% have PAE, 50% of these have ADHD-like symptoms, and 50% of these respond to TNS, then 222,750 children and their families could still obtain much needed relief in the US alone—and 100,000s more worldwide. Our imaging studies could, moreover, improve TNS by uncovering where it acts in the brain, which imaging measures are changed by it, and which portend favorable response. If, in contrast, TNS is not safe or effective for PAE, it will still be impactful to demonstrate to clinicians that PAE is a negative prognostic factor when treating patients with TNS.

Specific Aims

1. **Aim 1 Conduct a 4-Week Open Trial of Nightly TNS** (30/24 children pre/post-attrition aged 8-12 yr with PAE) **to test feasibility of TNS.** *We hypothesize TNS will be feasible (safe, well-tolerated, promising clinical efficacy) for pediatric PAE.*
2. **Aim 2 Acquire MRI, magnetic resonance spectroscopy (MRS), diffusion tensor imaging (DTI), and resting-state functional magnetic resonance imaging (rsfMRI) of the brain before and after TNS.** *In anterior cingulate cortex (ACC), middle frontal cortex (MFC), and inferior frontal cortex (IFC), we hypothesize that mean glutamate (Glu) levels will increase and functional connectivity (fc) will decrease after TNS. Pre-TNS, low local gyrification index (LGI), myelin, and Glu and high mean diffusivity (MD) and fc in these regions will predict favorable post-TNS response, including diminished hyperactivity/impulsivity, increased attention, and improved executive function.*

Hypotheses:

Aim 1: TNS will be feasible (safe, well-tolerated, promising clinical efficacy) for pediatric PAE.

Scores on all behavioral measures will move from the clinical to the nonclinical range. For example, a T score moving from 1 ½ standard deviations above the mean of 50 to less than 1 ½ standard deviations will indicate clinical efficacy

Aim 2: Mean Glu will increase and fc will decrease after TNS. Pre-TNS, low LGI, myelin and Glu and high MD and fc in ACC, MFC, and IFC will predict favorable post-TNS response.

Subjects: Prenatal alcohol exposure (PAE)

Inclusion:

Cohort 1: “PAE”

- Fetal alcohol syndrome, partial fetal alcohol syndrome, or alcohol-related neurodevelopmental disorder per modified Institute of Medicine criteria (thus positive maternal drinking in pregnancy required, facial stigmata not required)
- PAE >6 drinks/week for ≥2 weeks and/or ≥3 drinks on ≥2 occasions throughout gestation per Health Interview for Women/Health Interview for Adoptive and Foster Parents (HIW/HIAFP)
- Diagnosis of DSM-5 ADHD, including problems with inattention, hyperactivity, impulsivity, and/or executive function. Screening for ADHD will be done using the SNAP IV. Formal diagnosis of ADHD will be based on the MINI-KID with input from the Behavior Rating of Executive Function (BRIEF II) and the Conners 4.
- Parent and child able to complete testing in English
- Child able to cooperate during MRI
- Full-Scale IQ >70 per K-BIT-2
- Child able to comply with study procedures
- Age 8-12

Exclusion

- Other toxic exposure per HIW/HIAFP whose influence clearly surpasses that of alcohol (very rare) per study clinician (Drs. O’Connor, Schneider, or delegate) judgement
- Known genetic syndrome associated with ADHD-like symptoms including fragile X, tuberous sclerosis, or generalized resistance to thyroid hormone
- Serious medical or neurologic illness likely to influence brain function, e.g., seizures, closed-head trauma
- Gestation <34 weeks
- Ferromagnetic metal, claustrophobia, or other MRI or TNS contraindication (e.g., insulin pumps or other body-worn devices)
- Diagnosis of autism spectrum disorder, psychotic disorder, or major mood disorder
- Active suicidal ideation as evidenced by meeting criteria for “Current” or “Lifetime attempt” on the Suicidality module or “Current’ or ‘In early remission’ on the Suicide Behavior Disorder module of the MINI KID

Note on Medication

- Given frequent prescription of psychotropic medication in this population, recruiting exclusively unmedicated children is not feasible. Therefore, medication will be allowed in this study, but its use will be recorded. Patients currently on stimulant medication will be asked to withhold medication on assessment days.

The study team will make use of a checklist of Inclusion/Exclusion criteria when making the enrollment decision for each participant. The study team will also maintain a CONSORT-type diagram of participant screenings, exclusion, enrollment, retention, and dropout throughout the study. Data will specify reasons for study exclusion and dropout. Data from subjects excluded at screening will be fully anonymized; personal health information from these subjects will be destroyed.

Sample size: 30 PAE pre-attrition (24 post-attrition)

Subject identification and recruitment:

Subjects will be recruited through the Child and Adolescent General Outpatient Clinic, the Child Day-Treatment Program, and the Psychopharmacology Clinic in the Semel Institute. The Outpatient Clinic sees ~4000 children/year, ~ 5% of whom have PAE. Subjects will also be recruited from the UCLA Division of General Pediatrics at Mattel Children's Hospital, which sees 13,000 children/year. In addition to General Pediatrics, the Division of Genetics serves patients and families at risk for birth defects, developmental disabilities, and hereditary disorders. ~10% of children in these clinics have PAE. Beyond UCLA, we will recruit from practitioner referrals, schools, and the community using flyer posted at YMCAs, afterschool programs, barber and beauty shops. We also recruit from UCLA, FASD United, and FASD parent support websites. Social media will notify families of the study. We will use GoogleAds. We expect to enroll 30 patients (24 post-attrition) over 2 years.

Study Assessments

Screening and Eligibility Assessments:

Screening. Swanson, Nolan and Pelham (SNAP-IV) Rating Scale (Bussing et al. 2008)—This is a long-standing, very widely used scale with good validity and reliability for detecting ADHD and quantifying ADHD symptom severity in children and young adults. It is administered by a clinician or researcher to parents. Children enrolled in our study must have confirmed PAE with a diagnosis of ADHD as part of their presentation (this is very common). In our study the Study Coordinator or a trained Research Assistant will administer the SNAP-IV during telephone screening to a parent or guardian of each prospective child with PAE. Purpose is to detect potential ADHD in the child and to screen out children with negligible ADHD symptoms. The SNAP-IV is also used, together with other instruments, to establish a diagnosis of ADHD during the eligibility visit.

Diagnosis of ADHD. MINI KID Parent Version. The MINI KID Parent Version refers to the parent version of the Mini International Neuropsychiatric Interview for Children and Adolescents (MINI-KID), a structured diagnostic interview used to assess various mental health disorders in children and adolescents from 6 to 17 years by asking questions directly to the child's parent, providing insight into their observations of the child's behavior and potential symptoms, allowing for a more comprehensive evaluation compared to just interviewing the child alone. Responses to the MINI-KID are binary (yes/no), indicating the presence or absence of a psychiatric disorder. The test uses branching loci where further questions are asked only if the initial screening questions indicate a potential diagnosis. The MINI-KID has previously demonstrated strong psychometric properties in clinical and general populations (Duncan et al. 2018, Sheehan et al. 2010). When tested during its initial development, researchers found the MINI reliability was excellent on interrater measures and very good on retest measures.

PAE Physical Examination (Part of PAE Assessment)—A Clinician Investigator or trained delegate conducts a physical of the child including measurement of height and weight, head circumference, and palpebral fissure lengths, as well as evaluation of upper lip and nasal philtrum and further assessments. Purpose is to gather evidence relevant to making a diagnosis of an FASD (fetal alcohol syndrome, partial fetal alcohol syndrome, or alcohol-related neurodevelopmental disorder). A diagnosis is required to rule-in subjects for the study. Thus, this is a detailed exam that can lead to a diagnosis of an FASD, including fetal alcohol syndrome (FAS), partial fetal alcohol syndrome (pFAS), or alcohol-related

neurodevelopmental disorder (ARND). We have recently demonstrated that non-FASD specialist child psychologists can be trained to perform this examination reliably (O'Connor et al. 2022).

Facial Photos—The original approved protocol called for conventional 2D photography of each subject's face to help identify and document facial dysmorphia potential associated with PAE. We have now expanded this part of the PE Examination to include three 2D pictures and three 3D pictures of the face from the front, side, and at a ¾ angle. We have also done this in our recent study of PAE (NIAAA R01AA025066). There are established links between neurodevelopment and the FASD facial phenotype. Without prior knowledge of PAE, FAS diagnosis (Astley et al. 2004, Hoyme et al. 2016) relies on accurate identification of abnormal facial features, growth deficits and CNS problems. However, since the extent of facial abnormalities may be dose-dependent and more evident in severe cases or in older children, more effective detection and quantification of facial dysmorphology, especially for mild or moderate cases and at younger ages may be needed. Understanding how facial dysmorphology is associated with brain abnormalities could likewise inform diagnosis and provide opportunities for intervention. Further, if demonstrated as sufficiently sensitive, using more cost effective 2D rather than 3D facial imaging might provide a new level of accessibility for diagnosing FASD in at-risk populations. Here we propose to collect 2D and 3D facial photographs to assess the potential of computer-based dysmorphology in predicting responses to TNS treatment.

Health Interview for Women (HIW; O'Connor & Kasari 2000), Health Interview for Adoptive and Foster Parents (HIAFP; Quattlebaum & O'Connor 2013)(Part of PAE Assessment)-- Exposure to alcohol and other teratogens is assessed with the HIW or the HIAFP. These scales were developed by Co-I Dr. O'Connor and colleagues at UCLA and have been used routinely for many years here and elsewhere. The HIW and HIAFP are structured interviews measuring the child's PAE and prenatal exposures to caffeine, tobacco, marijuana, methamphetamine, cocaine, barbiturates, opioids, hallucinogens, anticonvulsants, antibiotics, and OTC painkillers. They are administered by a Clinician Investigator or trained delegate to the birth mother, foster parents, or other informant. Purpose is to rule-in children with sufficient *in utero* ethanol exposure to qualify for the study and to rule-out children with exposures to other teratogens that, in the opinion of a Clinician Investigator (Drs. O'Connor or Schneider), have impact exceeding that of ethanol (very rare).

Kaufman Brief Intelligence Test –Second Edition Revised (K-BIT-2)-- The K-BIT-2 is a brief screening tool used to assess intellectual functioning in individuals from 4 to 90 years (Kaufman & Kaufman 2004). The KBIT-2 IQ Composite score is an estimate of general intellectual functioning standardized with a mean of 100 and a standard deviation of 15. The IQ Composite score has high internal consistency across ages 4 through 18 (M = 0.93) with test-retest reliability of 0.88. The correlation between the IQ Composite score and the General Ability Index of the WISC-IV is 0.84. It is administered by a trained Research Assistant to the child. Purpose is to rule-out children with intellectual disability (ID; IQ <70).

Pubertal Development Scale (PDS, Petersen et al. 1988)—Given the long-observed secular trend towards earlier puberty in children and at the request of our NIAAA review panel, we will use the PDS to measure pubertal development. It is a physically non-invasive test. Given that most youth are unreliable completing this questionnaire, it is advised to administer the scale to parents. The PDS includes five items that ask about growth in height, body hair, and skin changes. The PDS also asks about breast development and menarche in females and deepening of voice and growth of hair on face for males. Participants rate each item on a 1 (barely started) to 4 (seems complete) scale. The PDS has demonstrated good internal consistency with Cronbach's α ranging between 0.91 and 0.96 and high test-retest reliability (ICC=0.81–0.92). Pubertal stage will be used as a covariate in statistical analyses.

Pre-Exposure to MRI Scanner—In a mock MRI scanner, children are given relaxation and breath training, and training in keeping still. From a distance, children are also shown the real MRI scanner. Purposes are pre-exposure for relief of anxiety and to rule-out children with claustrophobia or poor compliance.

Pre-TNS (Treatment) Assessments:

Clinical Global Impression—Severity (CGI-S) and Clinician's Global Impression—Improvement (CGI-I)(Guy 1976)—These extremely widely used, multiply validated 7-point scales assess initial severity and improvement in core symptoms of a chosen mental disorder (here ADHD) based on the clinician's experience with typical patients. They are administered by a Clinician Investigator, in this case to the parent reporting on the child. The CGI-S is administered at intake. Purpose is to serve as one metric of pretreatment severity of ADHD-like symptoms of PAE. The CGI-I is administered after TNS treatment and at follow-up. Purpose is to serve as one metric of post-treatment change in severity of ADHD-like symptoms.

ADHD-IV-Rating Scale (ADHD-RS; DuPaul et al. 1998)—The ADHD-RS is a parent interview administered by a Clinician Investigator. It was the primary outcome in the clinical trials leading to FDA-clearance of TNS for unmedicated pediatric ADHD. We use it to assess severity of ADHD-like symptoms in children with PAE at baseline and after the TNS treatment regimen.

Conners 4 Parent Version (Conners 4 P; Conners 2022)— The Conners 3 Parent Version originally proposed for this study to measure symptoms of ADHD is no longer available and has been replaced with the Conners 4 Parent Version (Conners 4 P) which provides a comprehensive assessment of symptoms and impairments associated with ADHD and common co-occurring problems and disorders in children and youth aged 6 to 18 years. The Conners 4 P is an inclusive and standardized measure that builds on the strengths of the Conners Rating Scales. Now fully digital, it provides online scoring for better data visualization, easy inventory management, a digital manual, and printable forms. It addresses critical concerns with Conduct and Self-Harm items and a Sleep Problems indicator. It also evaluates new content areas and common co-occurring problems such as Emotional Dysregulation, Depressed Mood, and Anxious Thoughts. Finally, the Conners 4 P provides a more comprehensive picture of how a rater approaches completing the measure, using new and updated validity scales, the number of omitted items, and the average number of items completed per minute. The Conners 4 P has excellent internal consistency (median omega coefficient = 0.94), strong test-retest reliability (median r = 0.89), and convergent validity (median r = 0.73). The parent completes the Conners 4 P before and after treatment.

Behavior Rating Inventory of Executive Function (BRIEF II) (Gioia et al. 2000)—The BRIEF II is a validated assessment of executive functions for children and adolescents that is very widely used clinically and in research. It is administered to parents before and after treatment by a Study Researcher. It takes 10-15 min. We use it to assess severity of executive function symptoms in children with PAE at baseline and after the TNS treatment regimen.

NIH Toolbox (Gershon et al. 2010)—The NIH Toolbox is a multidimensional set of measures to assess neurological and behavioral function available for clinicians and researchers. It is a valid, reliable, brief, and state-of-the-art instrument. We administer only the Cognition and Emotional Control Batteries to our pediatric patients. The latter assess domains of the PAE behavioral phenotype in DSM-5. (The primary author of this section of the DSM-5 is Co-I Dr. O'Connor.) The Cognition Battery yields Cognitive Function Composite, Fluid Cognition Composite (Dimensional Change Card Sort, Flanker Inhibitory

Control and Attention, Picture Sequence Memory, List Sorting, and Pattern Comparison measures), and Crystallized Cognition Composite (includes Picture Vocabulary and Reading Recognition measures) Scores. The Emotional Control Battery surveys Positive Affect, Emotional Support, Friendship, Loneliness, Perceived Rejection, Perceived Hostility, Self-Efficacy, Sadness, Perceived Stress, Fear, and Anger. The Toolbox is administered by the Study Coordinator to the child before and after the TNS treatment regimen. Purpose is to assess baseline and post-treatment changes in function in cognitive and emotional control domains.

Delis-Kaplan Executive Function System (D-KEFS) Verbal Fluency (Delis et al. 2001,2004)—Unpublished data from our recent study of PAE (NIAAA R01AA025066) show significantly inferior performance (p <0.001-0.041; effect sizes 0.4-1.2) on 7/9 tests of the D-KEFS Verbal Fluency assessment. This is consistent with many prior reports of verbal fluency deficits in PAE (Iosub et al. 2018, Kingdon et al. 2016, Kodituwakku et al. 2006, Kovács et al. 2023, Mattson & Riley 1999, Panczakiewicz et al. 2018, Rassmussen & Bisanz 2009, Schonfeld et al. 2001, Vaurio et al. 2008). There are indications that disability in verbal fluency is mediated by executive function deficits (Doyle et al. 2018). TNS, as mentioned elsewhere in this proposal, improves executive function deficits in children with ADHD. We will administer the D-KEFS Verbal Fluency assessment to determine whether TNS induces improvement in verbal fluency. The D-KEFS Verbal Fluency Test is comprised of three testing conditions: Letter Fluency, Category Fluency, and Category Switching. This test measures multiple aspects of verbal behavioral productivity and cognitive flexibility. It evaluates effectiveness of novel and semantic search strategies, and assesses flexibility in the implementation of semantic search strategies. The process approach enables further evaluation of self-monitoring of information search, as well as difficulties related to initiation and sustaining effort. There are three conditions in the Verbal Fluency test in which the examinee must say as many words as they can by letter, category, and category switching prompts. 1. The examinee says words beginning with a specified letter as quickly as possible; 2. The examinee is asked to say words belonging to a designated semantic category; and 3. The examinee must alternate between saying words from two different; semantic categories. The test will be administered by the Study Coordinator or SRA I to the child.

Test of Narrative Language-- A common Narrative Protocol in developmental research utilizes the wordless picture book Frog, Where Are You? (Mayer 1969; FWAY) to elicit narratives from children. The book illustrates a story in which a boy and dog set out to search for their missing pet frog, encountering numerous mishaps until they are reunited and return home. The story is fun, engaging, and provides material for the production of a rich narrative in 29 illustrated frames. The Protocol is designed to allow for the evaluation of the presence, correctness, and frequency of narrative elements such plotline identification (main plot and digressions), thematic identification (Miles & Chapman 2002), mean length of utterance (MLU), as well as for a wide range of linguistic elements: morphological errors, use of complex syntax, complexity of narrative structure, and types and frequency of evaluative devices. The Protocol is commonly used to study language development in typically and non-typically developing populations, such as children with Down's Syndrome (Miles & Chapman 2002); specific language impairment, early focal brain injury, Williams syndrome (Reilly et al. 2004), and fetal alcohol syndrome disorders (FASD; Vega-Rodríguez et la. 2020).

Procedure. The in-person interview is conducted by two adult experimenters. One of them acts as a listener for the child. The other introduces the storybook, gives instructions to the child, and audio-records the stories that the child creates. The child is asked to watch a video of the picture book silently (https://www.youtube.com/watch?v=BwDc3aOb-E0). Next the experimenter asks the child to narrate the picture book as they watch the video a second time. Administration takes ~6-8 min, further reducible (Berman & Slobin 1994,2013) if subjects are tired or insufficiently engaged.

The Revised Child Anxiety and Depression Scale (RCADS) for youth 8-18 years, is a 47-item, youth self-report questionnaire with subscales including: separation anxiety disorder, social phobia, generalized anxiety disorder, panic

disorder, obsessive compulsive disorder, and low mood (major depressive disorder). It also yields a Total Anxiety Scale (sum of the 5 anxiety subscales) and a Total Internalizing Scale (sum of all 6 subscales). Additionally, The Revised Child Anxiety and Depression Scale – Parent Version (RCADS-P) similarly assesses parent report of youth's symptoms of anxiety and depression across the same six subscales. One-week test-retest coefficients were good (Chorpita et al. 2000) with good concurrent validity compared to the Children's Depression Inventory and with the Revised Children's Manifest Anxiety Scale (Chorpita et al. 2005). The RCADS will be administered before and after treatment.

The Affective Reactivity Index (ARI; Stringaris et al. 2012) was developed to measure irritability in children from birth to 12 years. The ARI was specifically designed to obtain comparable information from youth and their parents. Using U.S.- and U.K.-based samples, the parent- and self-report forms of the ARI showed excellent reliability and formed a single factor. In terms of validity, the parent- and self-reported ARI total score successfully differentiated cases from controls in clinical and community samples. The parent-rated ARI total score also differentiated between youth with severe mood dysregulation and youth with bipolar disorder. Internal consistency: In the US sample, Cronbach's alpha was 0.92 and 0.88, for the parent- and self-report scales, respectively. Regarding construct validity, the ARI showed a gradation with irritability significantly increasing from healthy volunteers through to severe mood dysregulation (SMD). The ARI will be administered before and after treatment.

Children's Depression Inventory 2 (CDI 2; Kovacs 2010)-- The Children's Depression Inventory 2 contains 28 items, each of which consists of three statements. For each item, the child is asked to select the statement that best describes his or her feelings. The assessment is designed for multiple settings, including schools, child clinics, pediatric practices, and child psychiatric settings. The CDI 2 assesses self-reported key symptoms of depression, such as feelings of worthlessness and loss of interest in activities, and supports early identification and diagnosis of depressive disorders. Importantly, suicidality as a potential withdrawal criterion is included in the assessment. The CDI 2 will be administered to the child before and after treatment.

Magnetic Resonance (MR) Examinations of the Brain—In addition to evaluating feasibility of TNS treatment for PAE, this study explores possible brain bases of TNS therapy and predictors of clinical response. For these purposes, children undergo an ~60-min examination in a conventional clinical MRI scanner (3 T, 32-channel headcoil). Children are safety screened for MRI contraindications including metal in or on the body or clothing. Children's heads are firmly and comfortably positioned in the scanner with ample head padding. An MRI-opaque Vitamin E capsule is taped to the right scalp to ensure proper left-right lateralization of images in post-processing. Ear plugs are provided to protect against loud scanner noise. Patient and scanner operator have audio contact over a loudspeaker and microphone at all times. The patient also has an emergency squeezebulb to sound an alarm in case of distress. Multiple whole-brain scans are acquired, each in a different MR modality. They include rapid scout structural scans, eyes-open resting-state fMRI (rsfMRI), high-resolution structural MRI, echo-planar spectroscopic imaging (EPSI), and diffusion tensor imaging (DTI). All of these scans are equivalent from a human subjects and safety point-of-view. MRI is acquired by two trained Study Researchers and/or Research Assistants before and after the TNS treatment regimen.

In-Treatment Assessments (nightly):

For each overnight session of TNS, the parent makes an entry into the TNS Diary. Purpose is to encourage and to record adherence with the treatment program.

In-Treatment Assessments (weekly):

Children’s Sleep Habits Questionnaire (CSHQ; Owens et al. 2000)—This is a validated, reliable parent-completed test (~10 min) of a child’s sleep quality and sleep behaviors. It is completed by the parent during the pretreatment assessment and at the end of each week of treatment. The CSHQ is reviewed by a Study Researcher.

Side Effects Rating Scale and Adverse Events Inventory (McGough et al. 2015)—These instruments were used by Dr. McGough and UCLA colleagues to assess potential side effects of TNS and adverse events in the clinical trials that led to FDA-clearance of TNS as a treatment for unmedicated pediatric ADHD. (Side effects and adverse events were mild and few.) We have slightly modified the Side Effects Rating Scale Parent Version in order to provide a Side Effects Ratin Scale Child Version worded in a way more easily understood by children. An open-ended (i.e., non-pre-specified) side effects question has been added to the end of each scale. Both are completed during pretreatment assessment and at the end of each week of treatment in the presence (telephone, Zoom, or in-person) of a study investigator. The Side Effects Rating Scale Parent is completed by the parent. The Side Effects Rating Scale Child is read by the investigator to the child. The Side Effects Rating Scale is reviewed by a Study Researcher; the Adverse Events Inventory is reviewed by the Study Coordinator. Parents and children are generally advised to report serious emergent side effects or adverse events to the Study Coordinator or to a Clinician Investigator (Dr. Schneider) as soon as possible after occurrence.

Post-TNS (Treatment) Assessments:

Conners 4 P (see above)—The parent completes a final Conners 4 P at the end of treatment.

CGI-I (see above)-- A Clinician Investigator administers the CGI-I to the parent at the end of treatment.

ADHD-RS (see above)-- A Clinician Investigator administers the ADHD-RS to the parent at the end of treatment.

BRIEF II (see above)—A Study Researcher administers the BRIEF II to the parent at the end of treatment.

NIH Toolbox (see above)—The Study Coordinator administers the two Toolbox batteries to the child at the end of treatment.

Delis-Kaplan Executive Function System (D-KEFS) Verbal Fluency. As above.

Narrative. As above.

CDI 2. As above.

RCADS. As above.

ARI. As above.

MR Exam (see above)— Two Study Researchers and/or Research Assistants conduct an MRI session at the end of treatment.

Follow-Up Assessments:

CGI-I (see above)-- A Clinician Investigator administers the CGI-I to the parent 4 weeks after the end of treatment.

ADHD-RS (see above)-- A Clinician Investigator administers the ADHD-RS to the parent 4 weeks after the end of treatment.

Primary Study Device

Active TNS is administered nightly for 4 weeks by the parent/caregiver during sleep. Stimulation is performed using the Generation 1.0 Monarch® eTNS® System (NeuroSigma, Inc., Los Angeles), FDA-cleared for unmedicated pediatric ADHD. This device has been used safely and successfully in other prior and ongoing TNS trials at UCLA. The stimulator is worn on the child's pajamas or T-shirt and is attached with thin wires to disposable, silver-gel, self-adhesive patch electrodes. Parents apply patches across their child's forehead to provide bilateral stimulation of both V1 trigeminal branches for ~8 hr nightly. Patches are removed each morning. The active condition uses a 120-Hz repetition frequency, with 250-ms pulse width, and a duty cycle of 30 s on/30 s off. Current settings from 2-4 mA (range 0–10 mA) are established at baseline by titration, which identifies a stimulation level below the patient's subjective level of discomfort. Power is provided by 9-V Li rechargeable medical-grade batteries (iPower v.3), which are checked and replaced regularly. Patients are informed at a scripted presentation that "pulses may come so fast or so slowly that the nerves in the forehead might or might not detect a sensation." Each night parents turn on the device, press the "up" button until stimulation is uncomfortable or until the device reaches maximum current, and then press "down" to decrease it by 1 0.2-mA step. In active devices, the current to the patch is limited to a safe range (0-10 mA). Some patients may feel some sensation, which generally fades with time. The parent/caregiver maintains a TNS Diary to record adherence. The Study Coordinator also keeps in daily contact with the parent/caregiver for this purpose. Weekly, the parent/caregiver completes the BRIEF II, the Children's Sleep Habits Questionnaire (CSHQ; Owens et al. 2000), the Side Effects Rating Scale and Adverse Event Inquiry (McGough et al. 2015), and the Conners 4 Global Impression (Conners 4; Conners 1997), a parent-rated scale of ADHD severity. The CSHQ evaluates whether treatment (positively or negatively) affects the child's sleep. Improvement in sleep quality was a side-benefit of TNS in the ADHD open trial (McGough et al. 2015) and severely disturbed sleep is often seen in PAE. The Study Coordinator asks about any safety or tolerance issues and refers the child, if needed, to a Clinician Investigator for management.

The FDA-clearance for the Monarch TNS device covers only children with ADHD who are not currently taking ADHD medication. Children with ADHD associated with PAE are not specifically excluded by the labelling, nor are they specifically included. This proposal will test the TNS device in children with ADHD associated with PAE, some of whom will be taking concurrent ADHD medication. Based on our Initial Assessment, we do not anticipate additional risks due to medication and we will not ask parents to take their children off medication. Thus, TNS treatment, at least for children on medication, will be off-label. Therefore, rather than as "treatment with a n FDA-cleared device within an approved indication", present research is classified as "investigational use of an NSR device".

This is a feasibility study. One goal is to assess the tolerability of TNS treatment in children with ADHD and PAE. One key question here is whether children will remove the TNS device overnight unbeknownst to their parents? Studies of TNS treatment of pediatric ADHD at UCLA to date are encouraging in that there have *not* been issues with children removing the device in these studies. In principle, however, it could be different for children with ADHD and PAE. In our study, parents of each child are given a TNS Instructions and FAQ document at the beginning of treatment. Among several troubleshooting tips, parents are advised what to do if their child removes the device. If they catch it in the course of the night, they are advised to replace the device with fresh electrodes. If they do not become aware until the next morning, they are advised to note the incident in the TNS Diary and to contact the study team. When reapplying the device the next evening, parents are advised that putting a headband over the electrodes may help, especially if the child is a restless sleeper. Additionally, the parents should ensure that no lotions or creams, that may facilitate slippage, are applied to the skin under or around the electrode. This information is also given to parents verbally at time of TNS instruction.

Per the study design, *TNS compliance* is defined as the child wearing the device for 21 of 28 nights of treatment. Thus, non-compliance is failure to wear the device for 7 hours or more on 8 or more nights during the treatment regimen. In such cases, we will contemplate investigator-withdrawal of the child from the study. Among other factors, the decision to withdraw a child will be made based on interim scores on the Conners 4, i.e., does the patient appear to be getting better, despite partial non-compliance? In the TNS Instructions and FAQ document and on the ICF, parents are informed that their child may be withdrawn from the study if he or she repeatedly removes the device overnight or otherwise fails to comply with study procedures. Although we use the figure of 8 nights as an investigators' guidepost, we do not give a specific number in the TNS Instructions or ICF. This is because we feel that, if we did, some parents might think it is "okay" to skip 8 nights of treatment or might misreport the numbers of or fail to report incidences of removing the device for fear their child being withdrawn from treatment. For what we really want is to encourage 100% compliance and to work with families who are having difficulties reaching that benchmark.

We will tally compliance and numbers of subjects withdrawn. Efficacy statistics will be computed twice. We will compute them once for all patients, completers and withdrawn. Thereby, we will use last observation carried forward (LOCF) for cognitive and symptom scores of patients withdrawn. We will compute efficacy statistics again for completers only. The results of both computations are useful and relevant. The former mitigates against bias in rejecting non-responders. The latter tells what efficacy is if compliance is achieved, a reasonable goal in most cases.

The investigators will withdraw any participant from the study who, in the judgement of the Study Physician, experiences excessive risk (e.g., serious side-effects) or who requires prompt care (e.g., discovery of a serious incidental finding) that is incompatible with continued participation.

Generation 1.0 NeuroSigma Monarch® eTNS® System

Main component

Monarch (external pulse generator)

1. LCD Screen
2. Output Ports (CH1, CH2)
3. Up Key
4. Down Key
5. Lock Key
6. SELECT Key
7 ON/OFF Key
8. SET Key
9. Battery Cover

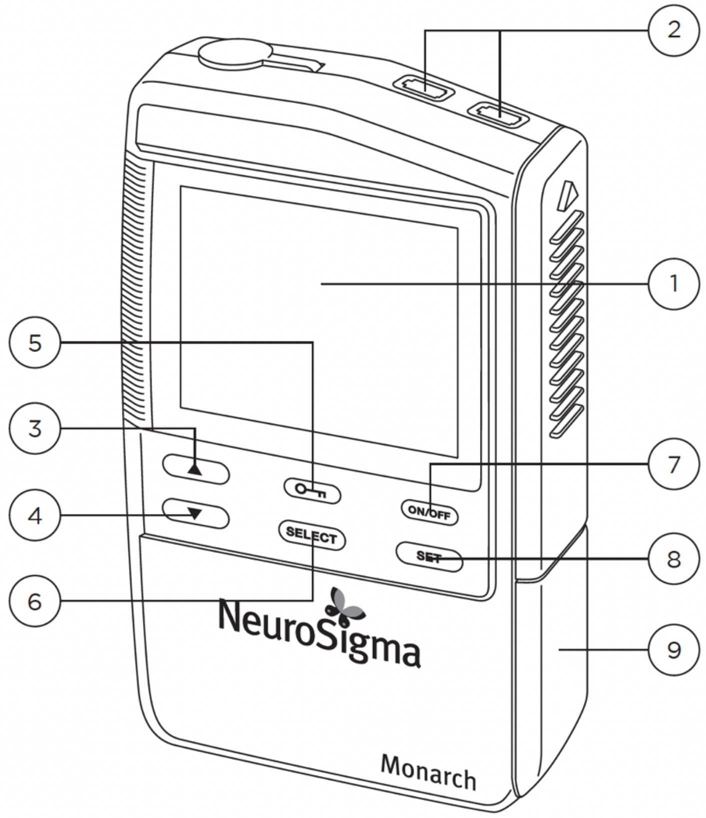

The Monarch eTNS System is designed to deliver electrical stimulation to the V1 branches of the trigeminal nerve, located just under the skin above the eyebrows, *via* a cutaneous electric patch.

The trigeminal nerve carries somatosensory information from the face to the brain. External trigeminal nerve stimulation (TNS) uses this pathway to send therapeutic stimulation into the brain. The trigeminal nerve connects from the face to the brainstem, and from there to other brain regions implicated in inattention, hyperactivity, and executive function deficits. While the exact mechanism of TNS is unknown, neuroimaging studies show that TNS increases activity in regions associated with regulating attention, emotion, and behavior.

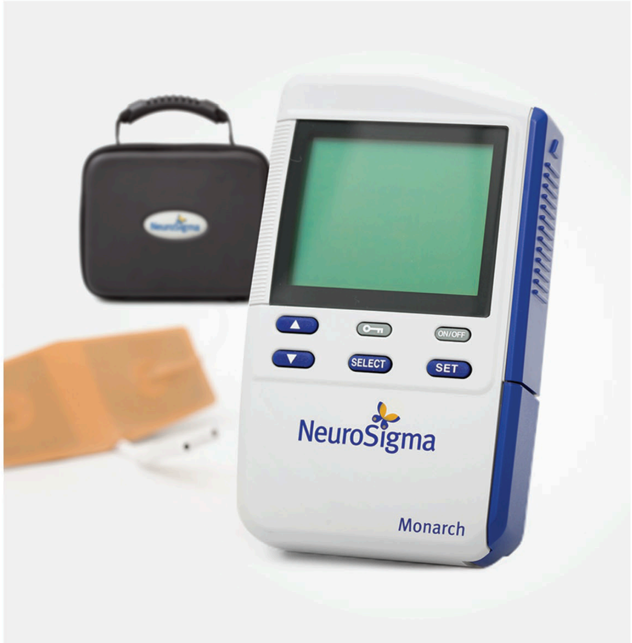

Back-Up Study Device

The Generation 2.0 Monarch® eTNS® System, also manufactured by NeuroSigma, is the back-up study device. By "back-up" we mean we intend to use the Gen 1 Monarch® for all 30 subjects for their full 4-week treatment regimens. The Gen 1, however, is being phased-out to be replaced with the Gen 2 starting in January 2025. We will be using brand-new Gen 1 devices. Nonetheless it is conceivable that one or more of our stock of Gen 1 devices will become unavailable or unusable due to damage, loss, theft, etc., and NeuroSigma will be unable to supply a new Gen 1 to replace it. Whenever this is the case, we will replace it with a Gen 2 in order to complete all or part of a patient's regimen. Therefore, we are requesting approval to use the Gen 2 as a therapeutically equivalent ersatz.

The brochure, manual, and FDA-clearance letter for the Generation 2.0 Monarch eTNS system are attached. The Gen 2 has nearly identical electrical parameters as the Gen 1. In particular, the Gen 2 delivers the same electric current and voltage stimulation waveforms to the patient. One small difference in electrical parameters between the two devices are that the Gen 2 has an output current range of 0–8 mA rather than 0–10 mA for the Gen 1. This was changed because few patients used the upper end of the range. The other small difference is that the Gen 2 output current is adjustable in increments of 0.1 mA, rather than 0.2 mA for the Gen 1. This was done to afford the patient, caregiver, or clinicain higher precision in adjusting the instrument. Rather than in electrical parameters, the Gen 2 mainly differs from the Gen 1 in having a slimmer form factor and user-friendlier software interface. If anything, these modifications render the Gen 2 even safer than the Gen 1.

The Gen 2 has the same FDA-clearance as the Gen 1. The PI has made an Initial Assessment that the Gen 2 device merits a classification as a non-significant risk (NSR) device. In this proposal we ask the IRB to agree or disagree with this classification. This IRB has already agreed with an NSR classification for the Gen 1 device.

Other study-related aspects of the Gen 2 are identical to those of the Gen 1 as described above.

Generation 2.0 NeuroSigma Monarch® eTNS® System

<table>
<tr><td>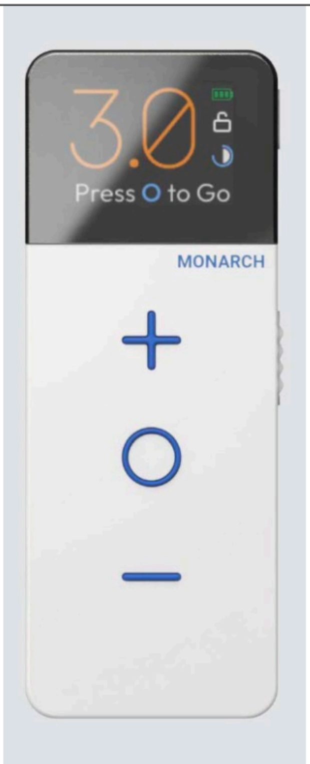
</td><td>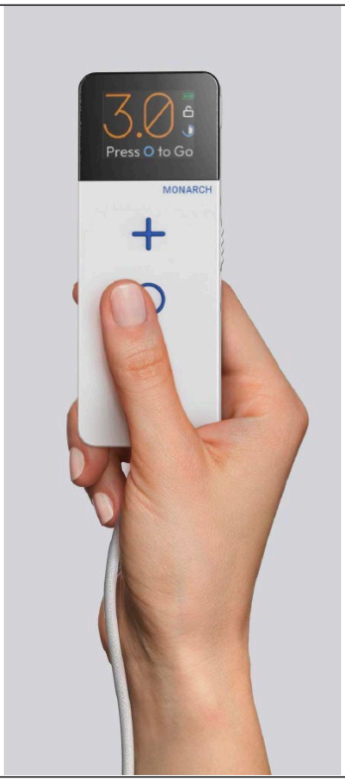
</td></tr>
<tr><td>A) The Monarch Gen 2.0 external pulse generator</td><td>B) B) The Gen 2.0 Monarch eTNS System held in a representative human hand for scale.</td></tr>
<tr><td colspan="2">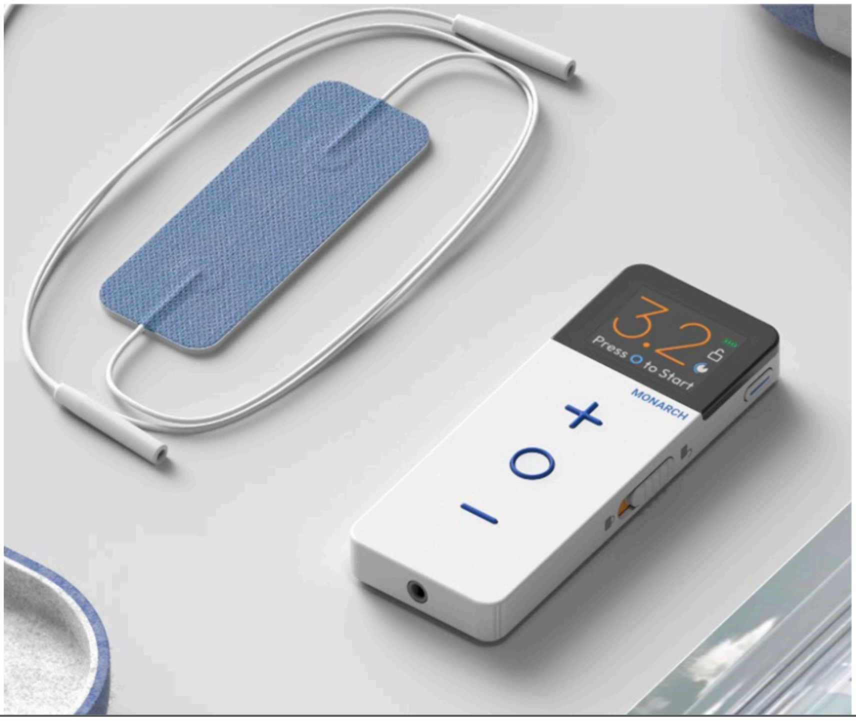
</td></tr>
<tr><td colspan="2">C) The Monarch NS-2 external (cutaneous) electrical patch beside the Gen 2.0 Monarch eTNS System.</td></tr>
</table>

Research Protocol:

1. Parents/caregivers of prospective patients undergo phone-screening in which:
    a. The Study Coordinator assesses likely presence of PAE, ADHD symptoms, and other eligibility criteria
    b. Assessment questions are asked from a prepared telephone screening script
2. Eligibility Assessment is performed for prospective patients passing screening:
    a. Parent consent and child assent are obtained
    b. Demographics, medical history, and current and past medications are collected
    c. Potential psychiatric comorbidities are diagnosed with the MINI KID, supported by clinical interview
    d. ADHD diagnosis is confirmed using the MINI-KID
    e. A K-BIT-2 IQ test is administered to the child
    f. Patients and their parent/caregiver undergo PAE Assessment. This includes a physical for the child to measure height, weight, and orbitofrontal circumference of the head and to identify potential facial and other stigmata of FASD. The HIW/HIAFP are structured interviews used to determine whether the biological mother of the child consumed ethanol and/or other teratogens while she was pregnant with the child and, if so, how much and how often (HIW). 3D photos are taken.
    g. Many children with PAE are raised in foster/adoptive settings. It is often not possible to contact the biological mother or she is unwilling to provide information about PAE. In such cases, we administer the HIAFP to a reliable caregiver familiar with the biological mother's history and/or solicit the child's medical records with the caregiver's permission, as these sometimes provide evidence of PAE. This is an accepted practice in FASD research
    h. Families with a child with PAE are informed of other treatment options
    i. The PDS is administered to evaluate puberty status of the child
    j. The child is exposed to a mock scanner and then child's ability to keep still is assessed
3. Pre-TNS assessment is performed for eligible patients
    a. Vitals of the child are taken
    b. The CGI-S and the ADHD-RS are administered by a Clinician Investigator to measure baseline ADHD symptoms
    c. The NIH Toolbox is administered by the Study Coordinator to probe the Cognition and Emotional Control behavioral domains
    d. The BRIEF II is administered to measure executive functions
    e. The Conners 4 is administered to measure inattention and impulsivity/hyperactivity
    f. The D-KEFS Verbal Fluency battery is administered to the child by the Study Coordinator
    g. The Test of Narrative Language is administered by the Study Coordinator to assay language impairments
    h. The CDI 2 is administered to assess depressive symptoms and possible suicidality as a withdrawal criterion
    i. The RCADS is administered to assess anxiety and depression symptoms
    j. The ARI is administered to assess irritability
    k. The Side Effects Rating Scale is administered to help determine whether any in-treatment side effects are device related
4. Pre-TNS MRI to be performed maximum 1 week after Pre-TNS assessment and maximum 1 week before commencing TNS treatment
    a. Multiple MR modalities are acquired: structural MRI, MRS (EPSI), DTI, and rsfMRI, all equivalent from a safety and human subjects perspective, in one 60-min session
    b. Throughout the brain, structural MRI is used to quantify cortical curvature and myelin content
    c. MRS is used to measure levels of Glu and other neurometabolites
    d. DTI is used to evaluate the microstructural properties of white matter
    e. rsfMRI evaluates fc
    f. A radiologist reads the pre-TNS structural MRI scans

    g. Subjects with findings of pathology in head or brain are informed and referred for treatment
    h. Significant pathology may result in exclusion of the patient from the study, whereby we consider that structural brain pathology sometimes attends PAE
5. TNS treatment with the Monarch® eTNS® System
    a. TNS treatment is described under Study Device above
    b. The Monarch is a small, portable, battery-powered unit. Thru two thin wires it delivers low-level current to a pair of electrodes in an adhesive patch on the forehead. The electrodes stimulate the V1 branch of the trigeminal nerve bilaterally
    c. The device is applied nightly by the child's parent/caregiver and provides stimulation for 7-9 hr overnight during sleep
    d. The parent/caregiver maintains a TNS Diary to ensure compliance, reinforced by daily telephone contact and weekly Zoom meetings with the Study Coordinator
    e. During the treatment regimen, the parent/caregiver performs Interim Assessments weekly of adverse-events (Adverse Events Inquiry), side effects (Side Effects Rating Scale), sleep quality (CSHQ), ADHD core symptoms (Conners 4) and executive function (BRIEF II)
    f. Each week, the Side Effects Rating Scale is administered by study staff to the child
    g. Each child undergoes 4 weeks of nightly TNS
6. Post-TNS Assessment in completers within 1 week of completing TNS; same assessments as Pre-TNS
    a. Height, weight, and vitals of the child are taken
    b. The CGI-I and the ADHD-RS are administered by a Clinician Investigator to measure baseline ADHD symptoms
    c. The NIH Toolbox is administered by the Study Coordinator to probe the Cognition and Emotional Control behavioral domains
    d. The BRIEF II is administered to measure executive functions
    e. The Conners 4 is administered to measure inattention and impulsivity/hyperactivity
    f. The D-KEFS Verbal Fluency battery is administered to the child
    g. The Test of Narrative Language is administered by the Study Coordinator to assay language impairments
    h. The CDI 2 is administered to assess depressive symptoms and possible suicidality as a withdrawal criterion
    i. The RCADS is administered to assess anxiety and depression symptoms
    j. The ARI is administered to assess irritability
    k.
7. Post-TNS MRI to be performed maximum 1 week after completing TNS treatment
8. 4 weeks after completing TNS, patients undergo Follow-Up Assessment, consisting of the CGI-I and the ADHD-RS.

Timeline Cohort 1:

Week	S	0	1-4	5	9
**Evaluations**	Demos/Hx/Rx<br>MINI-KID<br>HIW/HIAFP<br>ht/wt<br>PAE Ax and Photos<br>K-BIT-2 IQ<br>PDS<br>MRI Scanner Exposure	NIH Toolbox<br>BRIEF II<br>Conners 4<br>ADHD-RS<br>CSHQ<br>Side Fx RS<br>CDI 2<br>RCADS<br>ARI<br>D-KEFS VF<br>Narrative<br>vitals<br>CGI-S<br>TNS instruction<br><br>MRI	CSHQ<br>Side Fx RS<br>AE Inquiry	NIH Toolbox<br>BRIEF II<br>Conners 4<br>ADHD-RS<br>CDI-2<br>RCADS<br>ARI<br>D-KEFS VF<br>Narrative<br>ht/wt, vitals<br>CGI-I<br><br>MRI	CGI-I<br>ADHD-RS
**Treatment**			nightly TNS<br>TNS Diary<br>Zoom Conf		

Measurements:

1. Primary outcome: Score on the ADHD-RS, a clinician-rated measure of severity of core symptoms of ADHD; administered pre- and post-TNS
2. Secondary outcome: Score on the CGI-S/CGI-I ADHD, clinician-rated measures of severity of core symptoms of ADHD; CGI-S is administered pre-TNS and CGI-I is administered post-TNS
3. Magnetic Resonance Imaging: multiple modalities and endpoints, acquired pre- and post-TNS

Magnetic Resonance Imaging (MRI):

Magnetic Resonance Imaging (MRI) is performed by 2 Study Researchers at the UCLA Staglin IMHRO Center for Cognitive Neuroscience. Imaging is conducted using a 3-T whole-body scanner with a 32-channel phased-array head coil. Multiple MR modalities are acquired including structural MRI, rsfMRI, MRS, and DTI of the brain.

The Imaging Protocol includes the following sequences:

- Localizer
- rsfMRI
- T1w MPRAGE
- T2w MRI
- Echo-Planar Spectroscopic Imaging (EPSI) MRS
- DTI

All sequences are equivalent in their human subjects and safety aspects.

Data Analysis:

Mean differences are tested using the Linear Mixed Model in R with covariates and with last observation carried forward for early withdrawal patients. As needed, these include demographics (sex, age, IQ), other teratogen exposures (smoking, marijuana, illegal and prescription meds, caffeine), and clinical variables (DSM-5 comorbidities, medications). No correction for multiple comparisons is made for comparisons with *a priori* hypotheses (ACC, MFC, IFC). For other tests, FDR is applied. Efficacy will be computed twice. Efficacy will be computed once including all patients, both those who complete the study and those who were withdrawn using last observation carried forward (LOCF); efficacy will be computed again including completers.

Safety Monitoring plan:

Clinical Adverse Events

Clinical adverse events (AEs) will be monitored throughout the study.

There are no known risks of the MRI scanning procedures in this proposal. Some subjects may feel uncomfortable in the confined space of the scanner, and some individuals find the noise of the scanner bothersome. This is mitigated by pretraining of subjects in breathing and relaxation techniques and by mandatory use of earplugs. Subjects may experience increased anxiety before MRI scanning, as they may for any interview or medical examination. There is incidental risk in this study, which is the same incidental risk of being near any clinical MRI system, namely: (a) if a subject has an implanted electronic device (such as a pacemaker), it may stop working; (b) if a subject has implanted metal in the body (e.g., an aneurysm clip), it may become dislodged; and (c) certain metallic objects brought into the scanning room may be attracted to the magnet and thus there is the risk that these objects can become projectiles. These risks are mitigated by constant vigilance on the part of staff in prescreening subjects and in keeping ferromagnetic objects out of the scanner room. MRI risks are protected against by strict prescreening of patients, by use of ear protection, by acclimating patients to the scanner prior to scanning, and by adherence to safety protocols in the scanner room. While in the scanner, children are visually monitored by the scanner operator, operator and child are in contact over a speaker and a microphone, and the child has an emergency squeezebulb to sound an alarm for removal from the scanner in case of emergent distress.

Similar to MRI, contraindications of TNS include metallic implants, such as cardiac and neurostimulation systems. Dermatitis or sensitive skin represents a further contraindication. The Monarch device should not be used in patients with body-worn devices (e.g., insulin pumps or transcutaneous vagal nerve stimulators). There are also hazards with improper use of the device or attaching the patches other than on the forehead. Concurrent use of cellular telephones can interfere with TNS. We mitigate these risks thru thorough screening of patients and instruction in and monitoring of the use of the device. TNS risks are mitigated by preselection of patients, by thorough instruction in proper use of the device, and by persistent monitoring for adverse events.

TNS is generally well-tolerated. Relatively mild adverse events reported include:

- Bronchitis

- Headache

- Itching

- Light-headedness - Nausea

- Poor appetite - Skin rash

- Stomach ache

- Tooth pain

- Vomiting

- Trouble sleeping - Nightmares

- Drowsiness

- Fatigue

- Tingling

- Rapid heartbeat - Constipation

- Frequent urination - Increased appetite - Clenching teeth

The parent/caregiver maintains a TNS Diary to ensure compliance, reinforced by daily telephone contact with the Study Coordinator. During the treatment regimen, the parent/caregiver performs Interim Assessments weekly of adverse-events (Adverse Events Inquiry), side effects (Side Effects Rating Scale), sleep quality (CSHQ), ADHD core symptoms (Conners 4) and executive function (BRIEF II). The child completes the C-SSRS weekly to assess suicidality. The parent/caregiver and child are in daily contact with the Study Coordinator for reporting of any serious adverse events. The Study Coordinator promptly conveys such reports to a Clinician Investigator (Dr. Schneider or delegate). As part of completing the Adverse Events Inquiry and Side Effects Rating Scale, parents/caregivers monitor for such events and report them. The Clinician Investigator treats or refers such conditions for treatment as needed and decides whether or not discontinuing TNS is called for. These measures mitigate the risk of adverse events which overall is minimal and of low likely impact.

As detailed in the Initial Assessments for both the Gen 1 and Gen 2 Monarch® devices, we found no evidence for additional risks of TNS with concurrent medication. For the other major indications of TNS (depression, epilepsy, migraine) numerous clinical trials have allowed concurrent psychotropic medication and we found no serious adverse events associated with such concurrent use. The initial trials of TNS for ADHD were performed on unmedicated children, but the largest trial to date, the ATTENS trial in London, explicitly permits concurrent ADHD medication and we are aware of no serious adverse events in that trial either. TNS has generally been well tolerated with infrequent and mild side effects across its indications. Nonetheless, we will closely monitor for potential side effects and adverse events, including those associated with medication.

Adverse Event Reporting

When adverse events are detected, the Clinician Investigator treats or refers for treatment, as appropriate. TNS can also be discontinued at the discretion of the Clinician Investigator, if warranted.

Since the study device has significant safety data, significant AEs are not expected. If any unanticipated problems related to the research involving risks to subjects or others happen (including serious AEs) these are reported to the IRB. AEs that

are not serious but that are notable and could involve risks to subjects are summarized in narrative or other format and submitted to the IRB at the time of continuing review.

A radiologist reviews images after each MRI visit and provides a detailed report, including any incidental findings, to the Study Clinician (Dr. Schneider or delegate) and the PI. Patients with incidental findings of pathology, especially in head or brain on MRI, are informed and referred for further assessment and treatment.

Time and reimbursement for subjects:

Subjects receive a full course of an TNS treatment, clinician evaluations, and two brain MRI scans with readings at no cost to themselves or their families. In the R61 phase, families who complete the entire study will additionally receive $100 in the form of retail gift certificates. Families who withdraw their child early will receive gift certificates in an amount proportional to the fraction of the study completed. Families whose child is withdrawn by the investigators will receive the full $100 in gift certificates. Families additionally receive reimbursement for parking and sustenance in the form of drinks and snacks are provided on days of study visits to UCLA. In the subsequent, R33 phase of the study, if approved, which places higher demands on patients and families and has a larger budget, families will receive $350 compensation in addition to parking and sustenance.

Significance:

If this proposal is successful, TNS may be added to the clinician's armamentarium for managing PAE, a condition urgently in need of effective novel therapies. Additionally, this proposal may help decipher the currently unknown brain mechanisms of TNS, a therapy in use for multiple disorders. Findings of this study may help in identifying patients who are likely responsive or resistant to TNS or to modify TNS protocols themselves for greater efficacy.

Bibliography:

Astley SJ. Diagnostic Guide for Fetal Alcohol Spectrum Disorders: The 4-Digit Diagnostic Code (2004).

Berman RA, Slobin DI. Relating events in narrative: The cross-linguistic study of narrative structure (1994).

Berman RA, Slobin DI. Overview of linguistic forms in the frog stories. In: *Relating Events in Narrative* (pp. 109-126). Psychology Press (2013).

Berthele R. The many ways to search for a frog story: On a fieldworker's troubles collecting spatial language data. *Crosslinguistic approaches to the psychology of language. Research in the tradition of Dan Isaac Slobin*, 163-74 (2008).

Bussing R, Fernandez M, Harwood M, Wei H, Garvan CW, Eyberg SM, Swanson JM. Parent and teacher SNAP-IV ratings of attention deficit hyperactivity disorder symptoms: psychometric properties and normative ratings from a school district sample. Assessment 15(3),317–328 (2008).

Chorpita B.F, Yim LM, Moffitt CE, Umemoto LA, Francis SE. Assessment of symptoms of DSM-IV anxiety and depression in children: A Revised Child Anxiety and Depression Scale. Beh Res Ther 38,835-855 (2000).

Chorpita BF, Moffitt C, Gray J. Psychometric properties of the Revised Child Anxiety and Depression Scale in a clinical sample. Beh Res Ther 43,309-322 (2005).

Conners CK. Conners 4 Parent Version. Multi-Health Systems, Inc.; Toronto (2022).

Delis D, Kaplan E, Kramar J. Delis-Kaplan Executive Function System. Pearson Assessment; San Antonio, TX (2001).

Delis DC, Kramer JH, Kaplan E, Holdnack J. Reliability and validity of the Delis-Kaplan Executive Function System: An update. J Int Neuropsychol Soc 10,301-303 (2004).

Doyle LR, Moore EM, Coles CD, Kable JA, Sowell ER, Wozniak JR, Jones KL, Riley EP, Mattson SN, CIFASD. Executive functioning correlates with communication ability in youth with histories of heavy prenatal alcohol exposure. *Journal of the International Neuropsychological Soci- ety*, 24, 1026–1037 (2018).

Duncan L, Georgiades K, Wang L, Van Lieshout RJ, MacMillan HL, Ferro MA, …Boyle MH. Psychometric evaluation of the mini international neuropsychiatric interview for children and adolescents (MINI-KID). Psychological Assessment 30(7),916–928 (2018).

DuPaul GJ, Power RJ, Anastopoulos AD, Reid R. ADHD Rating Scale-IV Checklist, Norms and Clinical Interpretations. Guilford Press, New York (1998).

Gershon RC, Cella D, Fox NA, Havlik RJ, Hendrie HC, Wagster MV. Assessment of neurological and behavioural function: the NIH Toolbox. **Lancet Neurol 9**(2),138-139 (2010).

Gioia GA, Isquith PK, Guy SC, Kenworthy L. Test review behavior rating inventory of executive functioning. **Child Neuropsychol 6**,235-238 (2000).

Guy W. ECDEU assessment manual for psychopharmacology. US Department of Health, Education and Welfare publication (ADM) 76-338, 218–222. National Institute of Mental Health: Rockville, MD (1976).

Hoyme HE, Kalberg WO, Elliott AJ, Blankenship J, Buckley D, Marais A-S, Manning MA, Robinson LK, Adam MP, Abdul-Rahman O. Updated clinical guidelines for diagnosing fetal alcohol spectrum disorders. **Pediatrics** e20154256 (2016).

Iosub S, Fuchs M, Bingol N, Gromisch DS. Fetal alcohol syndrome revisited. **Pediatrics 68**,475–479 (1981).

Kaufman AS, Kaufman NL. Kaufman Brief Intelligence Test – Second Edition (K-BIT-2). American Guidance Service; Circle Pines, MN (2004).

Kingdon D, Cardoso C, McGrath JJ. Research Review: executive function deficits in fetal alcohol spectrum disorders and attention-deficit/hyperactivity disorder – a meta-analysis. **J Child Psychol Psychiatry 57**,116–131 (2016).

Kodituwakku PW, Adnans CM, Hay A, Kitching AE, Burger E, Kalberg WO, Viljoen DL. May PA. Letter and category fluency in children with Fetal Alcohol Syndrome from a community in South Africa. **J Studies Alcohol 67**,502-509 (2006).

Kovács MV, Lages YVM, Vieira BS, Charchat-Fichman H, Landeira-Fernandez J, Krahe TE. Neuropsychological evaluation of children and adolescents with fetal alcohol spectrum disorders in the Brazilian population. **Appl Neuropsych Child** DOI: 10.1080/21622965.2023.2279202 (2023).

Mattson S, Riley EP. Implicit and explicit memory functioning in children with heavy prenatal alcohol exposure. **J Int Neuropsychol Soc 5**,462–471 (1999).

McGough JJ, Loo SK, Sturm A, Cowen J, Leuchter AF, Cook IA. An eight-week, open-trial, pilot feasibility study of trigeminal nerve stimulation in youth with attention-deficit/hyperactivity disorder. **Brain Stim 8**,299-304 (2015).

McGough J, Sturm A, Cowen J, Tung K, Salgari GC, Leuchter AF, Cook IA, Sugar CA, Loo SK. Double-Blind, sham-controlled, pilot study of trigeminal nerve stimulation for ADHD. **JAACAP 58**(4),403–411 (2019).

Miles S, Chapman RS. Narrative content as described by individuals with Down syndrome and typically developing children (2002).

O'Connor MJ, Dillon A, Best KM, O'Neill J, Kilpatrick LA, Joshi SH, Alger JR, Levitt JG. Identification of seminal physical features of prenatal alcohol exposure by child psychologists. **J Ped Neuropsychol 8**,60-67 (2022).

O'Connor MJ, Kasari C. Prenatal alcohol and depressive features in children. **ACER 24**(7),1084-1092 (2000).

Owens JA, Spiritio A, McGuinn M. The Children's Sleep Habits Questionnaire (CSHQ): psychometric properties of a survey instrument for school-aged children. **Sleep 15**,1043-1105 (2000).

Panczakiewicz AL, Glass L, Coles CD, Kable JA, Sowell ER, Wozniak JR, Jones KL, Riley EP, Mattson SN, CIFASD. Neurobehavioral deficits consistent across age and sex in youth with prenatal alcohol exposure. **ACER 40**,1971–1981 (2016).

Kovacs M. Children's Depression Inventory, 2nd ed. Pearson Assessment; San Antonio, TX (2010).

Petersen AC, Crockett L, Richards M, Boxer A. A self-report measure of pubertal status: Reliability, validity and initial norms. **J Youth Adol 17**(2),117-133 (1988).

Quattlebaum JL, O'Connor MJ. Higher functioning children with prenatal alcohol exposure: Is there a specific neurocognitive profile? **Child Neuropsychol 19**(6),561–578 (2013).

Rasmussen C, Bisanz J. Executive functioning in children with fetal alcohol spectrum disorders: profiles and age-related differences. **Child Neuropsych 15**,201–215 (2009).

Reilly J, Losh M, Bellugi U, Wulfeck B. "Frog, where are you?" Narratives in children with specific language impairment, early focal brain injury, and Williams syndrome. *Brain and language*, *88*(2), 229-247 (2004).

Schonfeld AM, Mattson SN, Lang AR, Delis DC, Riley EP. Verbal and nonverbal fluency in children with heavy prenatal alcohol exposure. **J Stud Alcohol 62**,239–246 (2001).

Sheehan DV, Sheehan KH, Shytle RD, Janavs J, Bannon Y, Rogers JE, Milo KM, Stock SL, Wilkinson B. Reliability and validity of the Mini International Neuropsychiatric Interview for Children and Adolescents (MINI–KID). **J Clin Psychiatry 71**(3),313-326 (2010).

Stringaris A, Goodman R, Ferdinando S, Razdan V, Muhrer E, Leibenluft E, Brotman MA. The Affective Reactivity Index: a concise irritability scale for clinical and research settings. **J Child Psychol Psychiatry 53**(11),1109-1117 (2012)

Vaurio L, Riley EP, Mattson SN (2008) Differences in executive functioning in children with heavy prenatal alcohol exposure or attention-deficit/ hyperactivity disorder. J Int Neuropsychol Soc 14:119–129.

Vega-Rodríguez YE, Garayzabal-Heinze E, Moraleda-Sepúlveda E. Language development disorder in fetal alcohol spectrum disorders (FASD), a case study. *Languages*, *5*(4), 37 (2020).
