# Supplementary figures and images for: Trigeminal nerve stimulation (TNS) for children with attention deficit/hyperactivity disorder and fetal alcohol spectrum disorder: Feasibility study protocol
Source: PLoS One. 2025 Aug 29;20(8):e0330986. doi: 10.1371/journal.pone.0330986 (PMC12396707; doi:10.1371/journal.pone.0330986)

## Template CONSORT diagram

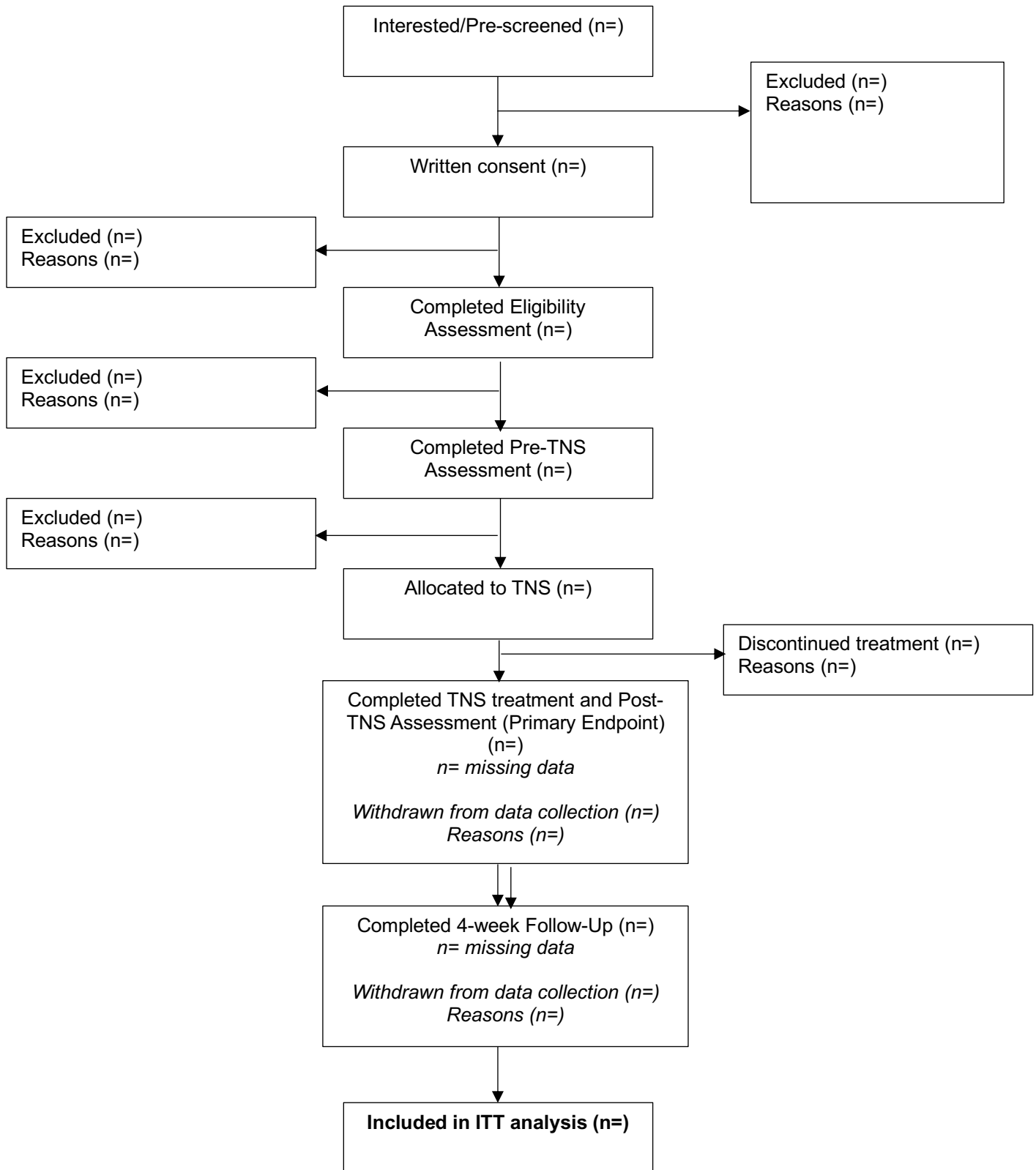

Supplement: S3 File — (PDF) [file pone.0330986.s003.pdf]
